# A New Approach for Interferent-Free Amperometric Biosensor Production Based on All-Electrochemically Assisted Procedures

**DOI:** 10.3390/bios15080470

**Published:** 2025-07-22

**Authors:** Rosanna Ciriello, Maria Assunta Acquavia, Giuliana Bianco, Angela Di Capua, Antonio Guerrieri

**Affiliations:** Dipartimento di Scienze di Base e Applicate, Università degli Studi della Basilicata, Via dell’Ateneo Lucano 10, 85100 Potenza, Italy; rosanna.ciriello@unibas.it (R.C.); maria.acquavia@unibas.it (M.A.A.); giuliana.bianco@unibas.it (G.B.); angela.dicapua@unibas.it (A.D.C.)

**Keywords:** biosensor, glucose biosensor, electrophoretic enzyme immobilization, electropolymerization, permselective polymers, electroanalytical chemistry

## Abstract

A new approach in amperometric enzyme electrodes production based on all-electrochemically assisted procedures will be described. Enzyme (glucose oxidase) immobilization was performed by in situ co-crosslinking of enzyme molecules through electrophoretic protein deposition, assuring enzyme immobilization exclusively onto the transducer surface (Pt electrode). Analogously, the poor selectivity of the transducer was dramatically improved by the electrosynthesis of non-conducting polymers with built-in permselectivity, permitting the formation of a thin permselective film onto the transducer surface, able to reject common interferents usually found in real samples. Since both approaches required a proper and distinct electrochemical perturbation (a pulsed current sequence for electrophoretic protein deposition and cyclic voltammetry for the electrosynthesis of non-conducting polymers), an appropriate coupling of the two all-electrochemical approaches was assured by a thorough study of the likely combinations of the electrosynthesis of permselective polymers with enzyme immobilization by electrophoretic protein deposition and by the use of several electrosynthesized polymers. For each investigated combination and for each polymer, the analytical performances and the rejection capabilities of the resulting biosensor were acquired so to gain information about their sensing abilities eventually in real sample analysis. This study shows that the proper coupling of the two all-electrochemical approaches and the appropriate choice of the electrosynthesized, permselective polymer permits the easy fabrication of novel glucose oxidase biosensors with good analytical performance and low bias in glucose measurement from typical interferent in serum. This novel approach, resembling classical electroplating procedures, is expected to allow all the advantages expected from such procedures like an easy preparation biosensor, a bi-dimensional control of enzyme immobilization and thickness, interferent- and fouling-free transduction of the electrodic sensor and, last but not the least, possibility of miniaturization of the biosensing device.

## 1. Introduction

Biosensors, i.e., “*analytical devices incorporating a biological material, a biologically derived material or a biomimic intimately associated with or integrated within a physicochemical transducer or transducing microsystem*” [1,2] (see Scheme S1), are well-known tools in analytical chemistry, designed, developed and used in a multitude of fields, e.g., for clinical and medical analysis and point-of-care testing (POCT). Developed almost 60 years ago by Clark and Lyons [3], biosensor research, production, and commercialization were mainly focused on those devices incorporating enzymes (mainly oxidoreductases) as biological material and using electrochemical detectors as transducers, the so-called enzyme amperometric biosensor [4].

The analytical performances of these devices, in terms of sensitivity, selectivity, and stability usage, come mainly from the choice of the enzyme, its immobilization approach onto the electrochemical transducer and from the selectivity of the transducer itself [5]. Indeed, while the selectivity, if not the specificity, of the target analyte detection could be assured from the right choice of the enzyme used in these devices and by a proper enzyme immobilization procedure, the overall sensing efficiency could be hampered by the amperometric detection, especially in complex samples like serum, where the presence of endogenous electroactive compounds (e.g., ascorbate) and surface active high molecular weight proteins (e.g., albumin) can severely interfere with the analyte detection (see Scheme S2). For these reasons, conventional enzyme amperometric biosensors are usually fabricated by using up to three membranes to address electrode fouling, enzyme immobilization, and electroactive interferences (see Scheme S3) [6]. Unfortunately, both cast and discrete membranes like these seriously limit the sensor fabrication to macroscopic devices, complicate their assembly, and lead to complex analyte diffusion with unusual responses and high response time.

While mediated amperometric enzyme electrodes [6], using synthetic mediators like ferrocene, almost solved the interference effects by lowering the detection potential, conversely, the enzyme entrapment in electrosynthesized polymers, the so-called “electrochemical immobilization” [7], can be considered an alternative tool for the realization of amperometric enzyme electrodes (see Scheme S4), allowing several important advantages such as an one-step preparation of biosensor, a bi-dimensional control of enzyme immobilization and thickness, miniaturization, and multilayer and/or multienzyme structures facilities. Particularly, the use of non-conducting polymers with built-in permselectivity, e.g., poly(o-phenylenediamine) [8], polyphenol [9], poly-2-naphthol [10], poly(o-aminophenol) [11], and overoxidized polypyrrole [12], resulted in an improved and significant sensor selectivity so to permit their application for real sample analysis. Nevertheless, several drawbacks afflict this powerful method. In fact, the electrostatic and/or chemical incompatibility of some enzymes and/or substrates with certain polymers and the chemical conditions required for the electrosynthesis [7,13], the need of a significant, non-denaturing adsorption of the enzyme onto the electrode surface for a successful immobilization [14,15] which in the best cases involves a low amount of immobilized enzyme (up to few monolayers), and the modification of the permselectivity behavior due to polymer ageing [16,17], all contribute to reduce the performances of these biosensors.

The “*hybrid*” approach (see Scheme S5), coupling the advantages of electrosynthesized, non-conducting polymers with those of classical enzyme immobilization methods, apparently seems to overcome the above problems. In this respect, the commonly used immobilization method can be considered the co-crosslinking of the enzyme with an inert protein such as bovine serum albumin (BSA) through a crosslinker such as glutaraldehyde (GLU) [18]. As examples, the immobilization of glucose oxidase (GOD) onto a platinized RVC electrode by crosslinking with GLU, followed by electropolymerization of 1,2-diaminobenzene [16] or 1,3-diaminobenzene and 1,3-dihydroxybenzene [17] permitted the development of a glucose biosensor displaying satisfactory anti-interferent characteristic whose lifetime was limited by the stability of the immobilized enzyme instead of the polymer film. On the basis of a co-crosslinking procedure already studied and optimized (Ref. [19] and references therein), the proper coupling of a overoxidized polypyrrole-modified platinum electrode with GOD immobilized by GLU co-crosslinking with BSA resulted in a glucose biosensor [19] with high enzyme loading, good shelf lifetime, and wide linear range; because of the excellent interferent rejection of overoxidized polypyrrole (interference bias in the low micro molar range). The sensor has been successfully applied for glucose determination of untreated serum samples from both normal and diabetic subjects. As a proof of the effectiveness of this method, this “*hybrid*” approach was also investigated and applied to other enzymes like choline oxidase and acetylcholinesterase [20], l-lysine-α-oxidase [21], and alkaline phosphatase [22], developing novel biosensors which proved successful even in real sample analysis. Nevertheless, the enzyme immobilization procedure used in this approach, although effective, needs precise casting of the enzyme crosslinking solution onto the electrode surface and so it is limited to macroscopic electrodes of simple geometries, for example. Of course, if this enzyme immobilization procedure could be carried out to induce co-crosslinking exclusively onto the electrode surface, as is known to happen, e.g., for electroplating, surely these drawbacks could be surmounted.

In a recent paper from our laboratory [23], an original method for enzyme immobilization, we call “*electrophoretic protein deposition assisted* in situ *co-crosslinking enzyme immobilization*”, almost promising for the above purposes, was developed and deeply studied. Here, the well-known electrophoretic deposition (ED) [24,25], where charged particles (ions or surface charged colloids) migrate, permitting their deposition onto the surface of an electrode of opposite charge, was applied to proteins and enzymes, thus realizing what is known as electrophoretic protein deposition (EPD).

**Scheme 1:**
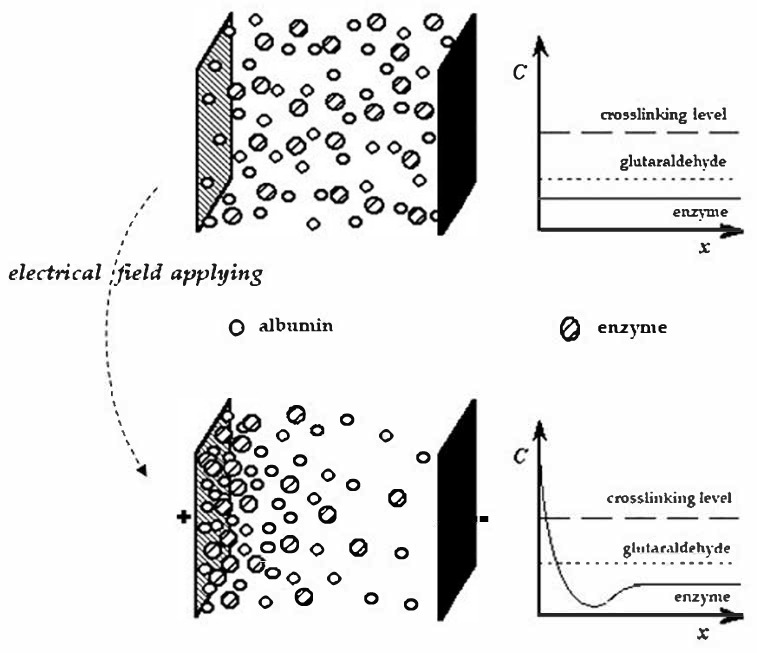
Schematization of the EPD process referring to the electrophoretic protein deposition assisted in situ co-crosslinking enzyme immobilization used. The large rectangle couples in each left panel represent the electrodes, each with its proper charge. Circles and ellipses between electrodes for each left panel represent the albumin protein and the enzyme molecules, respectively, while each right panel shows their concentrations near the electrode surface when the electrical field is applied (**lower panel**) or not (**upper panel**) between electrodes.

As Scheme 1 shows, EPD was performed in a solution containing the enzyme of interest (GOD in the present case), BSA as inert protein, and GLU as crosslinker, all at low concentrations to significantly decelerate the co-crosslinking course during the time of the deposition experiment. The following application of the electrical field, causing the electrophoretic migration of GOD and BSA towards the electrode of opposite charge, produces a high increase in their concentrations at the electrode/solution interphase so to trigger an in situ co-crosslinking, solely onto the surface of the electrode. This novel approach, combining the benefits of the classical enzyme immobilization techniques to those inferred from a well-known electrochemical technique like electroplating (e.g., EPD), permitted [23] an accurate spatial control of the deposition process, whatever the dimension and shape of the deposition electrode, while simple parameters, such as the applied voltage, current used to generate the required electrical field, and the deposition time, could control and change the thickness of the enzyme deposit.

In this study, this original EPD approach was coupled to the electrosynthesis of polymers with built-in permselectivity to produce novel interferent-free amperometric glucose biosensors, all-electrochemically assisted assembled. Indeed, several biosensors using EPD for enzyme immobilization were already described ([23] and references therein) as well as biosensors incorporating electrosynthesized permselective films, e.g., [11], but the use and the proper coupling of these two electrochemically assisted procedures were never explored and reported. In this study, in fact, several strategies in biosensor fabrication were developed, studied, and described, particularly regarding the steps used for the EDP production of the enzyme membrane (here GOD) as well as for the in situ electrosynthesis of the permselective polymers; in this respect, several polymers coming from different monomers were electrosynthesized and tested to evaluate the best experimental conditions to produce this sensing devices. This study will show that this novel strategy in enzyme immobilization properly coupled with electrosynthesized permselective films is able to produce interference-free glucose biosensors with analytical performances that are surely promising for their application to real sample analysis, thus corroborating the effectiveness of the proposed all-electrochemically assisted procedures for biosensor assembling.

## 2. Materials and Methods

### 2.1. Materials

Glucose oxidase (EC 1.1.3.4., type VIIS, from *Aspergillus niger*, 162 units/mg solid), bovine serum albumin (fraction V), glutaraldehyde (grade II, 25% aqueous solution), and β-D-glucose were purchased from Sigma (Sigma Chemical Co., St. Louis, MO, USA). Potassium ferricyanide and pyrrole were obtained from Carlo Erba (Milano, Italy). The monomers *o*-aminophenol and *o*-phenylenediamine were purchased by Fluka (Fluka Chemie, Buchs, Switzerland). The interferents ascorbic acid, uric acid, acetaminophen (4-acetamidophenol or paracetamol), L-cysteine, and the monomer 2-naphtol were all obtained from Aldrich (Aldrich-Chemie, Steinheim, Germany). Hexaammineruthenium (III) chloride came from Johnson Matthey (Johnson Matthey GmbH, Sulzbach, Germany). All other chemicals were of analytical reagent grade. The water used for washing procedures and for preparing solutions was freshly first deionized then bidistilled before its use. The procedures for pyrrole, 2-naphtol, *o*-phenylenediamine, and *o*-aminophenol purification was described elsewhere [8,10,11,12]. Stock glucose solutions were made and stored as described elsewhere [8]. The dilute glucose solutions as well as the monomer and interferent solutions were prepared just before their use.

### 2.2. Apparatus

The potentiostat/galvanostat setup used for EPD enzyme immobilization has been described elsewhere [23]. Electrochemical experiments for biosensor studies and characterization were performed by using an AMEL (Milan, Italy) mod. 466 polarographic analyzer connected to a Linseis (Linseis GmbH, Selb, Germany) LY 18100 X-Y chart recorder. A conventional three electrode system with a Pt rod as counter electrode and an Ag/AgCl, KCl satd. reference electrode was utilized in all the electrochemical experiments. A platinum disk (polycrystalline 99.95%, 3.0 mm diameter, Goodfellow, Cambridge, UK) embedded in a glass body was used as the working electrode for the electrochemical studies. An EDI 101 rotating platinum disk electrode (2.0 mm diameter) connected with a CTV101 Speed Control Unit (both from Radiometer, Copenhagen, Denmark) was utilized for biosensor assembly and characterization. In EPD experiments involving enzyme immobilization, the working and the counter electrodes were assembled face to face to ensure a uniform electrical field near the working electrode. Before each experiment, the platinum disk working electrodes were cleaned and electrochemically treated as described elsewhere, e.g., [11]. All EPD, electrochemical, and biosensor experiments were performed at room temperature.

### 2.3. Polymer Electrosynthesis Deposition Procedure

The electrosynthesis of polymers from 2-naphtol, *o*-aminophenol, and *o*-phenylenediamine was performed by cyclic voltammetry in a 5 mM solution of the relevant monomer in phosphate buffer (pH 7, *I* 0.1 M). The scan rate was 50 mV/s, while the scan range was −0.1 ÷ 0.8 V, −0.1 ÷ 0.9 V, and −0.1 ÷ 0.6 V vs. Ag/AgCl satd. for polymers electrosynthesized from 2-naphtol, *o*-aminophenol, and *o*-phenylenediamine, respectively. Usually, for bare Pt, 20 scans were required to obtain a passivating polymer film, fully covering the electrode surface of the working electrode. In the case of Pt working electrodes already modified with an enzyme layer, the polymer electrosynthesis through the enzyme membrane required more scans (greater than 20) and higher deposition times (typically 20 min) for an efficient and complete polymer deposition.

The electrosynthesis of polymer from pyrrole was performed at a constant potential in a solution of pyrrole 0.4 M in KCl 10 mM. The applied potential was 0.7 V vs. Ag/AgCl satd. while the deposition charge was 300 mC/cm^2^. After that, the modified electrode was overoxidized at 0.7 V vs. Ag/AgCl satd. in a phosphate buffer (pH 7, *I* 0.1 M) overnight.

### 2.4. EPD Procedure for Enzyme Immobilization

Enzyme immobilization was typically performed by immersing the electrode (bare or modified by the chosen polymer) in a phosphate buffer solution (pH 7, *I* 0.1 M) containing a proper amount of proteins and crosslinker: unless otherwise stated, glucose oxidase was 0.5% *w*/*v*, bovine serum albumin 1% *w/v,* and glutaraldehyde 2.5% *v*/*v*. EPD and in situ co-crosslinking of glucose oxidase and bovine serum albumin onto the electrode surface was performed by a galvanodynamic deposition technique [23], using a pulsed current sequence of 2 mA/cm^2^ with a period of 5.5 s, a duty cycle of 9.1% (see Scheme S6 were *t*_1_ 5 s, *t*_2_ 0.5 s and *I* 2 mA/cm^2^), and a deposition time of 1 h, corresponding to an immobilized enzyme layer of about one micron thickness [23].

### 2.5. Biosensor Fabrication and Characterization

Two different approaches were investigated in biosensor fabrication which differ in the order of the deposition procedure. One consists of the preliminary polymer deposition onto the bare Pt working electrode followed by the EPD process for enzyme immobilization onto the polymer-modified electrode. The other required the preliminary enzyme immobilization onto the bare Pt working electrode by EPD followed by the electrosynthesis of the polymer onto the enzyme-modified electrode. After fabrication, the biosensors were thoroughly washed with water and stored in the dark at 4 °C in a phosphate buffer (pH 7, *I* 0.1 M).

Biosensor characterization was performed in air saturated stirred solutions using the above-described Pt-modified rotating disk electrode by injecting the desired aliquots of substrate or interferent solutions. Unless otherwise stated, the rotation rate was 1000 rpm.

## 3. Results and Discussion

### 3.1. EPD and in Situ Co-Crosslinking of Glucose Oxidase and Bovine Serum Albumin onto the Electrode Surface

Using electrophoretic deposition (ED) or electrophoretic protein deposition (EPD), only charged molecules, particles, or colloids can migrate under the applied electrical field and hence deposit onto the desired electrode. The proteins involved in EPD for enzyme immobilization, i.e., glucose oxidase (GOD) and bovine serum albumin (BSA), have their isoelectric points at pH 4.3 and 4. 7, respectively [26]. Accordingly, EPD was carried out in neutral solutions (i.e., phosphate buffer pH 7, *I* 0.1 M), and since both were in their negatively charged forms, they were attracted to the anode of EPD cell.

The method used for GOD enzyme immobilization by EPD was co-crosslinking [18,27], i.e., an inert protein-assisted enzyme crosslinking, which is known to minimize the side effects from classical crosslinking like high molecular crowding which adversely affects the catalytic activity of the immobilized enzyme. In fact, the use of BSA, for example, as an inert protein extensively reduces GOD crowding, giving rise to an immobilized enzyme layer with proper and optimal catalytic activity, as it will be shown later in the study.

To achieve successful in situ co-crosslinking of GOD and BSA by EPD, just onto the electrode surface, the kinetic of co-crosslinking reaction was drastically slowed down by lowering GOD and BSA concentrations in the bath solution to prevent any gelation process in the cell during the deposition procedure (i.e., even for several hours). The GOD and BSA levels at the anode of the EPD cell increased so high during the EPD process to yield co-crosslinking solely onto the electrode surface of the anode (see Scheme 1). The concentration levels of GOD, BSA, and GLU that guaranteed any co-crosslinking process in the cell but allowed enzyme immobilization under proper EPD conditions were those already described in the relevant Materials and Methods subsection, which agree with those already reported elsewhere [23]. Note that both galvanodynamic and potentiodynamic EPD deposition techniques proved successful for in situ enzyme immobilization [23]. In this study, the galvanodynamic approach was preferred due to the higher control of the enzyme layer thickness [23]. The experimental conditions used permitted the formation of an immobilized enzyme layer of about one micron thickness onto the electrode surface [23].

### 3.2. Permselective Behavior of the Enzyme Layer as Deposited by EPD

Since the main goal of this study was the production of novel biosensors where the enzyme immobilization step and the formation of permselective layer by electrosynthesized polymers were both electrochemically assisted to address the formation of these layers only and exclusively onto the electrode surface, the permselective behavior of the enzyme layer as deposited by EDP was preliminary investigated. Indeed, the presence of a proteinaceous layer on the electrode surface is expected to affect the electrochemical behavior of electroactive species, which should interact with and diffuse through the layer.

For this preliminary investigation, the electrochemical behavior of well-known electrochemical probes like ferricyanide and hexaammineruthenium (III) chloride, which are known both to show reversible electron transfer on Pt, were tested at bare Pt and Pt-modified electrodes with an enzyme layer. As Figure S1 shows, the electrochemical behavior of the ferro/ferricyanide couple was almost suppressed with the presence of the enzyme layer onto the electrode; on the contrary, the opposite behavior was observed for the electrochemistry of hexaammineruthenium (III) chloride where the cyclic voltammograms were almost the same at both the electrodes. Since both probes have a similar molar volume but opposite charge, this study showed that the presence of the enzyme layer involves mainly charge exclusion effects rather than size ones, in agreement with the charge distribution expected for a BSA-GOD co-crosslinking deposit (*vide ante*).

### 3.3. Study of the Coupling of the Electrosynthesis of Permselective, Non-Conducting Polymers with Enzyme Immobilization by EPD

Unfortunately, the permselective behavior of the enzyme layer towards negatively charged compounds is not sufficient to allow its application to real sample analysis, so the use of electrosynthesized non-conducting polymers, with built-in permselectivity, properly coupled with in situ co-crosslinking of GOD and BSA by EPD, was adopted and investigated. In this study, several permselective polymers, namely polyphenol (PP) [9], poly-*o*-aminophenol (PoAP) [11], poly-*o*-phenylenediamine (PoPD) [8], poly-2-naphtol (P2NAP) [10], and overoxidized polypyrrole (oPPy) [12] were used and tested, both as a permselective membrane as well as for their facility to be coupled with in situ co-crosslinking of GOD and BSA by EPD. As preliminary proof of their permselective properties, Figure S2 compares the electrochemistry of ferricyanide at bare and modified Pt electrodes with some permselective polymers. As can be seen, the expected electrochemical behavior is almost suppressed at electrodes modified by the electrosynthesis of these polymers, suggesting the formation onto the electrode surface of a passivating continuous film, free of holes or defects, permitting the permeation of ferricyanide. Conversely, the anodic limit of these modified electrodes, where water discharge and dioxygen evolution are expected, compares well with that of bare Pt (see Figure S3), showing that smaller molecules like water can almost permeate through the film and electrochemically react.

The assembly of biosensors based on both these electrochemically assisted deposit formations can obviously be realized in two different, opposite modes, depending on the nature of the first and second layer deposited onto the electrode surface. For simplicity and sake of clarity, those biosensors produced by direct electrosynthesis of the polymer onto bare electrode followed by in situ co-crosslinking of GOD and BSA by EPD were specified here as Pt/polymer/GOD electrodes; on the contrary, those obtained by in situ co-crosslinking of GOD and BSA by EPD on bare electrodes followed by electrosynthesis of the polymer will be identified here as Pt/GOD/polymer. Except for the order of the layers, these two approaches cannot be considered simply additive but reversed since their electrochemically assisted deposition processes could of course modify the underneath first layer. For example, the current pulses used in the galvanodynamic technique for the EPD-assisted enzyme immobilization could significantly modify the nature and the permselective properties of the bottom electrosynthesized polymer in Pt/polymer/GOD production as, e.g., observed for polypyrrole where its properties changed from conducting to insulating polymer after potential application [12]. Likewise, the presence of the underneath enzyme layer in Pt/GOD/polymer electrodes may hamper the electrosynthesis of the polymeric layer due to diffusional and/or electrostatic constraints between the monomer molecules and the enzyme layer.

**Figure 1:**
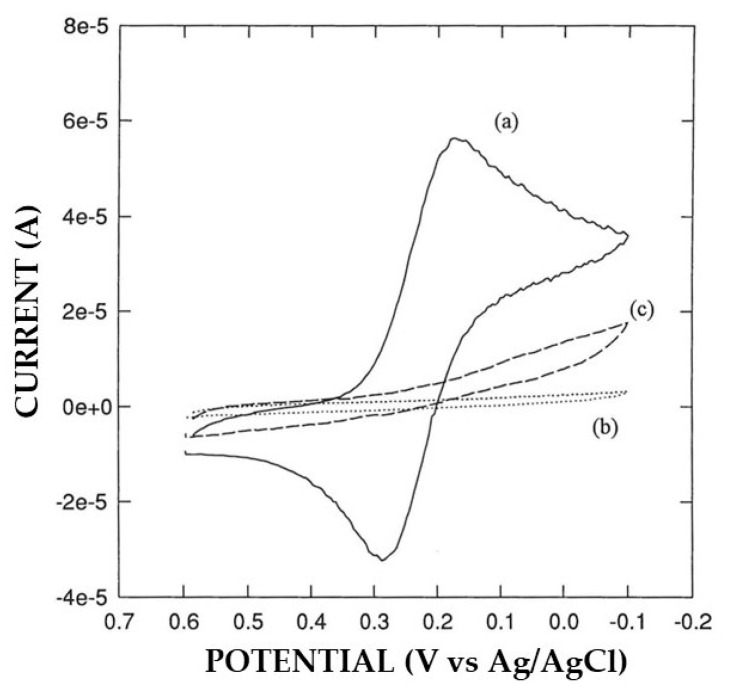
Typical cyclic voltammograms of potassium ferricyanide 5 mM in phosphate buffer (pH 7, *I* 0.1 M) on bare Pt electrode (a) and Pt-modified electrodes with poly-2-naphtol (P2NAP) before (b) and after (c) the current pulse application for EPD enzyme immobilization. The scan rate was 50 mV/s and the electrode diameter 3 mm; other conditions as described in the Materials and Methods section.

**Figure 2:**
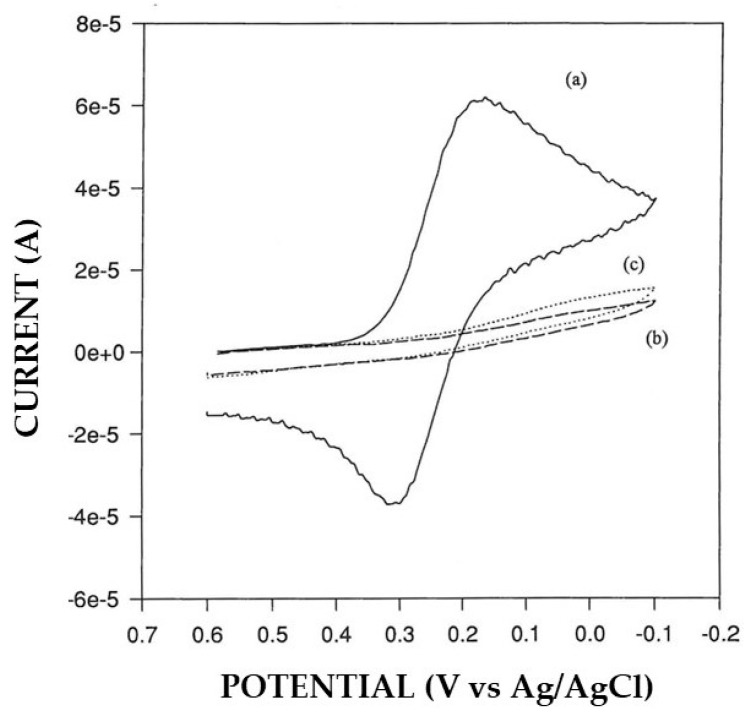
Typical cyclic voltammograms of potassium ferricyanide 5 mM in phosphate buffer (pH 7, *I* 0.1 M) on bare Pt electrode (a) and Pt-modified electrodes with poly-o-aminophenol (PoAP) before (b) and after (c) the current pulse application for EPD enzyme immobilization. The scan rate was 50 mV/s and the electrode diameter 3 mm; other conditions as described in the Materials and Methods section.

**Figure 3:**
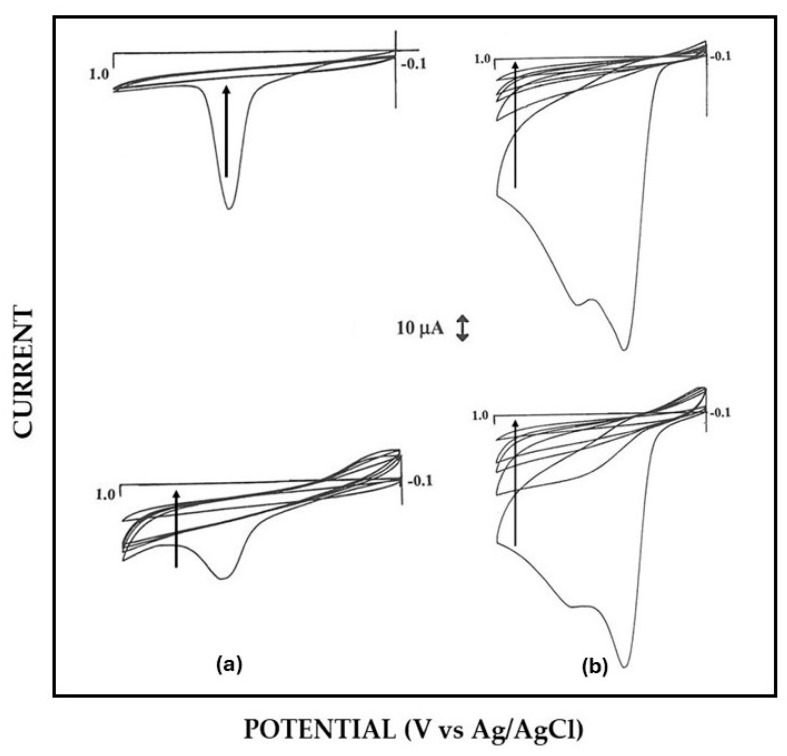
(**a**) Left panels—Typical cyclic voltammograms relevant to the electrosynthesis of poly-2-naphtol (P2NAP) from a solution of 2-naphtol 5 mM in phosphate buffer (pH 7, *I* 0.1 M) on bare Pt electrode (**upper panel**) and Pt-modified electrode by in situ co-crosslinking of GOD and BSA by EPD (**lower panel**). (**b**) Right panels—Typical cyclic voltammograms relevant to the electrosynthesis of poly-o-phenylenediamine (PoPD) from a solution of o-phenylenediamine 5 mM in phosphate buffer (pH 7, *I* 0.1 M) on bare Pt electrode (**upper panel**) and Pt-modified electrode by in situ co-crosslinking of GOD and BSA by EPD (**lower panel**). In all panels, the arrows show the time course of scan cycles. The scan rate was 50 mV/s and the electrode diameter 3 mm; other conditions as described in the Materials and Methods section.

As anticipated above, a significant alteration in permselectivity of the electrosynthesized non-conducting polymers was observed in Pt/polymer/GOD production in some cases. For example, Pt/P2NAP electrodes showed a definite lower permselectivity after current pulse application during the EPD-assisted enzyme immobilization succeeding step (see Figure 1). Further, the quality of proteic deposit was lower since it was highly spongy and easily detachable after electrode washing. A similar drop in permselectivity was observed also for other polymers (e.g., oPPy), but not for the case of Pt/PoAP-modified electrodes, where permselectivity did not change significantly (see Figure 2). On the other hand, the permselectivity observed for Pt/GOD/polymer electrodes was comparable to their Pt/polymer counterparts, showing that the electrosynthesis of these films through the enzyme layer was effective as well. In this respect, Figure 3a compares the cyclic voltametric profiles for P2NAP electrosynthesis at bare Pt and Pt/GOD electrodes. As can be seen, apart the lower kinetics in the deposition process and the consequential lower peak currents and small shift towards higher peak potentials, the electrochemistry of 2-naphtol during Pt/GOD/P2NAP production was almost suppressed after few scans as for bare Pt, demonstrating the formation of a non-conducting film and hence the feasibility of the approach. A similar lowering in the kinetics of polymer formation was also observed for other monomers, as Figure 3b shows for the production of Pt/GOD/PoPD electrodes from o-phenylenediamine, confirming the anticipated diffusional constraints of the monomer through the enzyme layer. As Figure 3b also confirms, the lower diffusion hampered only the kinetics of film formation onto Pt/GOD electrode but not the effectiveness of permselectivity performances of the produced polymer.

In agreement with these experimental findings, the fabrication of Pt/GOD/polymer sensors through these coupled electrochemically assisted deposit arrangements appeared more effective to produce interferent and fouling-free biosensors. Further investigation regarding the analytical performances of these biosensors was performed to consolidate these preliminary results.

### 3.4. Analytical Characterization of Pt/Polymer/GOD Biosensors

As already mentioned, the current pulses train utilized for the EPD-assisted enzyme immobilization somewhat modified the underlying film during the fabrication of Pt/polymer/GOD sensors, typically to such an extent to lower significantly their permselectivity. Since this was not the case at least for Pt/PoAP/GOD electrodes, they were fully characterized in terms of their analytical performances.

**Figure 4:**
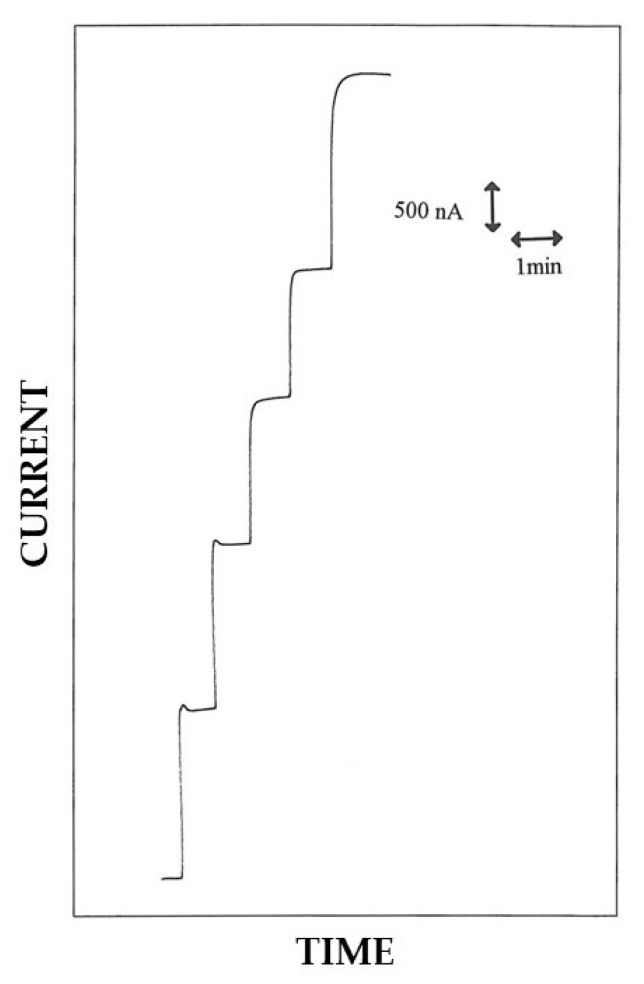
Typical current–time responses at a rotating disk Pt/PoAP/GOD electrode for sequential additions of glucose to an air-saturated phosphate buffer (pH 7, *I* 0.1 M). Glucose adding in figure refers to total glucose concentrations of 1.25, 2.49, 3.72, 4.95, and 7.39 mM. The disk rotation rate was 1000 rpm and the electrode diameter 2 mm; other conditions as described in the Materials and Methods section.

In this respect, Figure 4 shows typical current–time responses for successive additions of glucose standard solutions at a Pt/PoAP/GOD electrode in rotating disk mode. As can be seen, quick responses to glucose were observed, whose timing was almost limited by the mixing process in solution. The response time *t*_0.95%_, evaluated as the time required to reach the 95% of the maximal steady state current, was about 4 s in most cases. Figure S4 (upper plot) reports the relevant calibration curve displaying the linear and saturated response at low and high glucose concentrations, respectively, as expected for enzyme catalysis. A significant deviation from the Michaelis–Menten model usually adopted for enzyme catalysis was also observed (compare data points with Michaelis–Menten fitting) as confirmed by the relevant Eadie–Hofstee graph (Figure S4, lower plot) which improperly deviates from the expected linear behavior. Deviations like this are not unusual and have already been observed for several biosensors using co-crosslinking, such as the enzyme immobilization approach [19]. Accordingly, a more complex kinetic behavior is expected for the present biosensor where diffusion of substrate through the enzymic layer becomes limiting as well (*vide infra*).

Rotating disk electrodes are well-known to be a powerful tool for investigating and controlling mass transfer effects in electrochemistry, so a hydrodynamic study of the present rotating disk Pt/PoAP/GOD electrode was performed to obtain further insight on its kinetic behavior. In this respect, Figure S5 shows the normalized current responses of the biosensor due to the adding of glucose 1.5 mM in solution as observed at different rotation rates. As can be seen, a response increase was observed with the rotation rate, which reached a steady state value from ca. 1000 rpm, as expected for a membrane electrode [28] (like the present) under diffusion control. In fact, the diffusion current *i*_d_ for a rotating disk membrane electrode can be described as following [28]:

*i*_d_ = *i*_L_ [1/(1 + *P*_s_/*P*_m_)] (1)

where *i*_L_ is the current of a rotating disk electrode in absence of any membrane, i.e., the Levich current [29], while *P*_s_ and *P*_m_ are the permeability of solution and membrane, respectively, where *P*_s_ = *D*/δ_d_ (*D* the diffusion coefficient in solution and δ_d_ the diffusion layer thickness in solution), and *P*_m_ = α*D*_m_/δ_m_ (α the partition coefficient of solute in membrane, *D*_m_ the diffusion coefficient in membrane and δ_m_ the membrane thickness). At low rotation rates *P*_s_/*P*_m_ << 1, so [28]:

*i*_d_ = *i*_L_ = 0.62 *n F π r*^2^ *D*^2/3^ ν^−1/6^ ω^1/2^ *C* (2)

where *r* is the electrode disk radius, ν the kinematic viscosity, ω the rotation rate, *C* the bulk solute concentration, while *n* and *F* have the usual meanings. Therefore, *i*_d_ increases with rotation rate at low rates in agreement with the trend observed in Figure S5. Conversely at higher rates *P*_s_/*P*_m_ >> 1 and the current is [28]:

*i*_d_ = *i*_L_ *P*_m_/*P*_s_ = *n F π r*^2^ *P*_m_ *C* (3)

Hence, it is independent of rotation rate, but limited by membrane permeability, e.g., its thickness (*P*_m_ = α*D*_m_/δ_m_), as observed in Figure S5 at higher rotation rates. All these features shown in Figure S5 and their agreement with the expected membrane electrode behavior [28] demonstrated the diffusional constraints of the biosensor and its departure from the supposed Michaelis–Menten behavior. Please note that the behavior depicted in Figure S5 is entirely reversible, i.e., the steady-state current responses decreased when decreasing the rotation rate of disk electrode after reaching the higher rates shown in Figure.

Table 1 summarizes some of the analytical performances of the present Pt/PoAP/GOD electrode. As shown, the biosensor showed a good sensitivity and a linear range, eventually permitting glucose analysis even in a hyperglycemic sample when applied to real sample.

**Figure 5:**
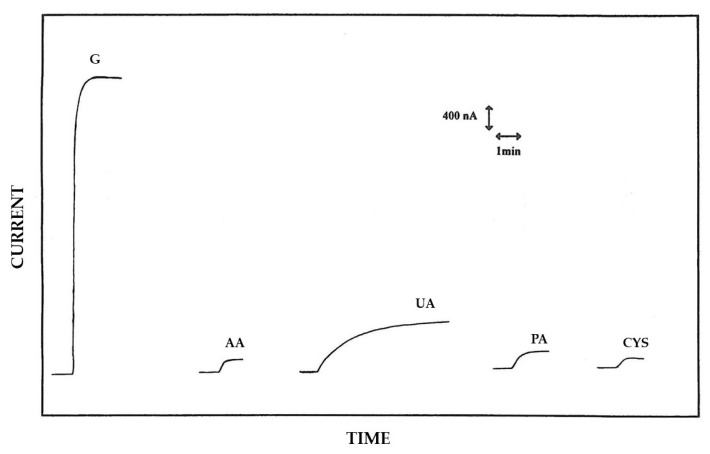
Typical current–time responses at a rotating disk Pt/PoAP/GOD electrode for additions of glucose (G) 5 mM, ascorbic acid (AA) 0.1 mM, uric acid (UA) 0.5 mM, paracetamol (PA) 0.2 mM, and cysteine (CYS) 0.1 mM to an air-saturated phosphate buffer (pH 7, *I* 0.1 M). The disk rotation rate was 1000 rpm and the electrode diameter 2 mm; other conditions as described in the Materials and Methods section.

Figure 5 compares a typical glucose response at 5 mM level as observed at a rotating Pt/PoAP/GOD electrode with those relevant to some representative endogenous serum interfering compounds like ascorbic acid (AA), uric acid (UA), and cysteine (CYS), at concentrations near the upper limits of their respective physiological concentration ranges. The response of paracetamol (PA), a typical exogenous interfering compounds used in this study as a representative drug, was evaluated and compared as well. In this respect, Table 2 summarizes the relevant bias introduced in glucose measurement from these representative interfering compounds. As can be seen from Figure 5 and Table 2, uric acid caused the higher bias in glucose measurements with higher values than the other interfering species. As it will be shown later in this study, the high interference observed for uric acid was not due to the poor permselective characteristics of PoAP film (which instead proved highly [11]), but, as anticipated above, somewhat from the electrical stress of PoAP film due to the successive EPD-assisted enzyme immobilization.

The storage stability of the present biosensor was examined by discontinuously monitoring, over more than a month, the glucose sensitivities of a typical Pt/PoAP/GOD electrode stored in a phosphate buffer (pH 7, *I* 0.1 M) at 4 °C in the dark when not in use, without using any efforts to avoid bacterial growth in the storage buffer. As Figure S6 shows, after an initial drop in sensitivity, the biosensor still showed a sensitivity of about 70% of its initial value for about 40 days, demonstrating a good stability of its use. Lastly, repeatability and reproducibility of the present biosensor were evaluated by the within-day (*n* = 3) and the between-days (*n* = 10) coefficient of variations for glucose response at 5 mM level which were 4% and 17%, respectively.

### 3.5. Analytical Characterization of Pt/GOD/Polymer Biosensors

As pointed out above, biosensors obtained by in situ co-crosslinking of GOD and BSA by EPD on bare Pt electrodes followed by electrosynthesis of the polymer on these enzyme-modified electrodes, i.e., the Pt/GOD/polymer biosensors, are expected to show an improved selectivity towards interferences. Indeed, the current pulses, used in the galvanodynamic technique for the EPD-assisted enzyme immobilization, which have proved to modify significantly the nature and the permselective properties of the electrosynthesized polymers, were applied well before their electrosynthesis for this biosensor assembly. To confirm this, a Pt/GOD/PoAP biosensor was studied and their analytical performances compared to the previous Pt/PoAP/GOD electrode.

**Figure 6:**
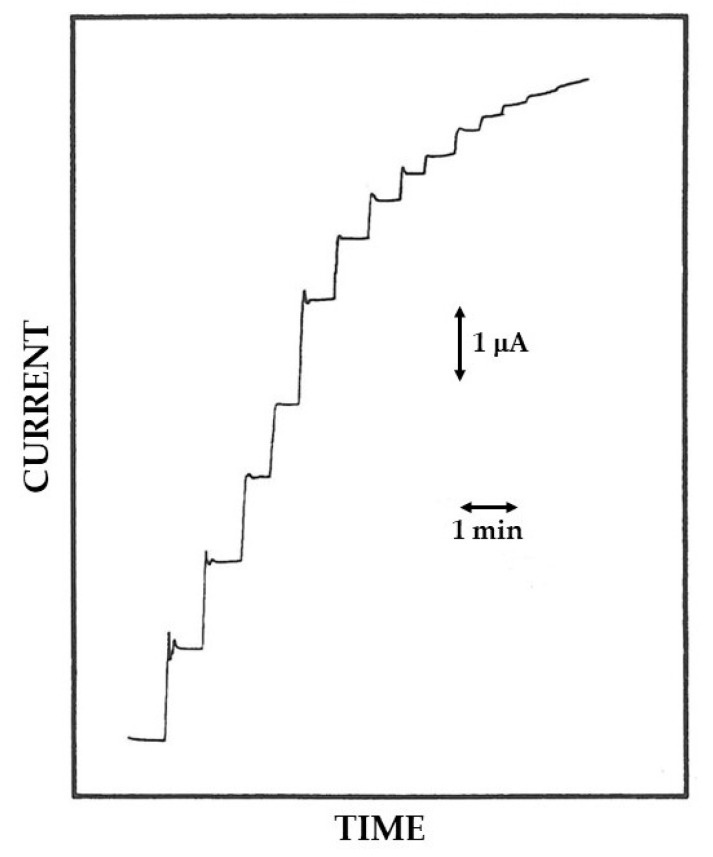
Typical current–time responses at a rotating disk Pt/GOD/PoAP electrode for successive additions of glucose to an air-saturated phosphate buffer (pH 7, *I* 0.1 M). Each response in the figure refers to total glucose concentrations of 1.25, 2.49, 3.72, 4.95, and 7.39 mM and more. The disk rotation rate was 1000 rpm and the electrode diameter 2 mm; other conditions as described in the Materials and Methods section.

Figure 6 shows some typical current–time responses observed at a Pt/GOD/PoAP electrode working in rotating disk mode for successive additions of glucose standard solutions. Fast current–time responses were observed also in the present case whenever the glucose levels; the response time *t*_0.95%_ was evaluated as nearly 5 s for this biosensor and compared well with the previous case. In this respect, it is worth noting that a brief response time represents a fundamental requirement for biosensor applications in flow analysis or in vivo monitoring.

Figure S7 (upper plot) reports a typical calibration curve for glucose at a Pt/GOD/PoAP electrode showing the expected behavior for enzyme catalysis. Also, in the present case, the relevant Eadie–Hofstee graph (Figure S7, lower plot) displayed a significant departure from the linear behavior expected for the classical Michaelis–Menten model, but the deviation was more striking, suggesting further diffusional constraints in its kinetic behavior. This complex kinetic behavior was also confirmed by a relevant hydrodynamic study reported in Figure S5. Also, for Pt/GOD/PoAP electrodes, the first portion of the curve shows an increase in the response with the rotation rate until reaching a steady state value at ca. 800 rpm, as expected for a membrane-modified electrode under diffusion control [28]. All the theoretical considerations and the relevant discussion mentioned above for the Pt/PoAP/GOD biosensor can be applied here as well.

The analytical performances of the present Pt/GOD/PoAP electrode are reported in Table 1. Notably, both sensitivity and maximal current were approximately half of the previous case, even if the present Pt/GOD/PoAP and the previously studied Pt/PoAP/GOD biosensors were made by equal layers but simply reversed. The exposure of the Pt/GOD electrode to the oAP monomer bath for successive electrosynthesis of PoAP layer may probably have been somewhat detrimental to GOD activity (*vide infra* for a further support of this hypothesis). Finally, the apparent Michaelis–Menten constant and the upper limit of the linear range were lower than the previous case, corroborating the important diffusional constraints in its kinetic behavior. Glucose sensing in the present case should require at least sample dilution for hyperglycemic analysis.

**Figure 7:**
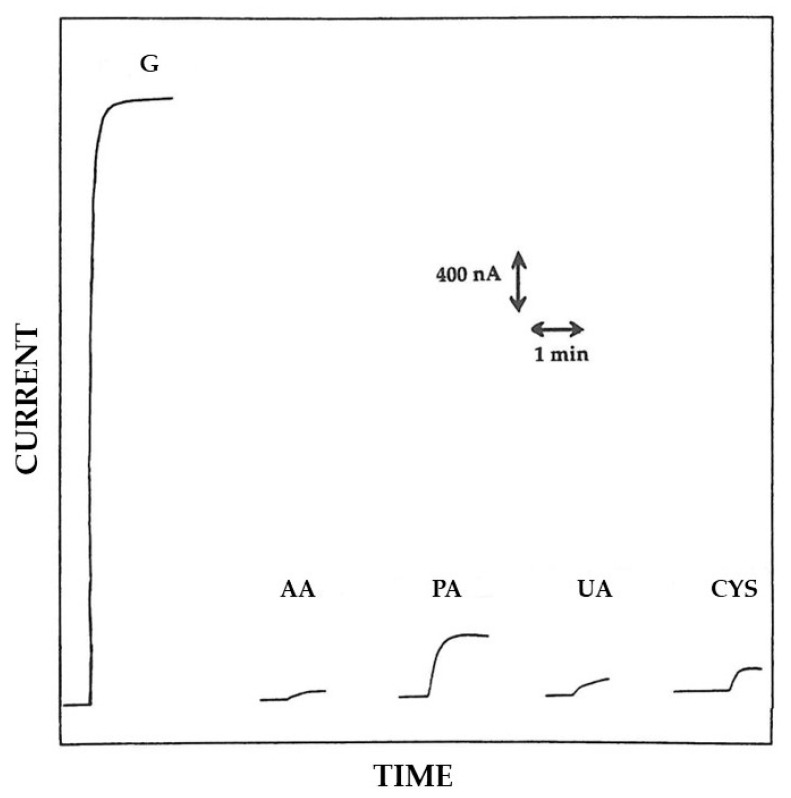
Typical current–time responses at a rotating disk Pt/GOD/PoAP electrode for additions of glucose (G) 5 mM, ascorbic acid (AA) 0.1 mM, uric acid (UA) 0.5 mM, paracetamol (PA) 0.2 mM, and cysteine (CYS) 0.1 mM to an air-saturated phosphate buffer (pH 7, *I* 0.1 M). The disk rotation rate was 1000 rpm and the electrode diameter 2 mm; other conditions as described in the Materials and Methods section.

An investigation of the analytical performances in terms of interference rejection ability has also been performed for this Pt/GOD/PoAP biosensor (see Figure 7) and Table 2 summarizes the relevant bias introduced in glucose measurement from the selected interfering compounds already tested before. As anticipated, the different approaches in biosensor production were beneficial since, e.g., the bias in glucose measurements from uric acid dropped significantly and that from ascorbic acid was even lower (see Figure 7 and compare bias in Table 2). With respect to the Pt/PoAP/GOD case, the bias from cysteine was almost identical (instead reducing) and that from paracetamol surprisingly doubled. This unexpected comportment could be evidence that the electrosynthesis of permselective film like PoAP through a proteic membrane (i.e., Pt/GOD) was not effective as in absence of any membrane on the electrode surface [11], thus leaving the question if this lower anti-interference performance should be expected even for other permselective films or likely represents a singular case.

Finally, Figure S8 shows the results of a storage stability study for the present Pt/GOD/PoAP electrode. For this biosensor, a satisfactory storage stability was observed since a sensitivity of about 80% of its initial value was still retained after about two weeks of storage and use. Moreover, the within-day (*n* = 3) and the between-days (*n* = 7) coefficient of variation for glucose response at 5 mM level was 6% and 20%, respectively.

To further explore and confirm the ability of proposed procedure in biosensor fabrication as well as to check if the electrosynthesized permselective films partially lost their anti-interference performances, a further biosensor based on the use of poly-2-naphtol (P2NAP) [10], namely a Pt/GOD/P2NAP, was developed and tested. Electrosynthesized films from 2-naphtol (2NAP) were chosen for their extreme rejection ability [10], thus permitting the analysis of a well-known neurotransmitter like acetylcholine and its metabolite choline in media rich in electroactive neurotransmitters like brain tissue homogenates [20].

**Figure 8:**
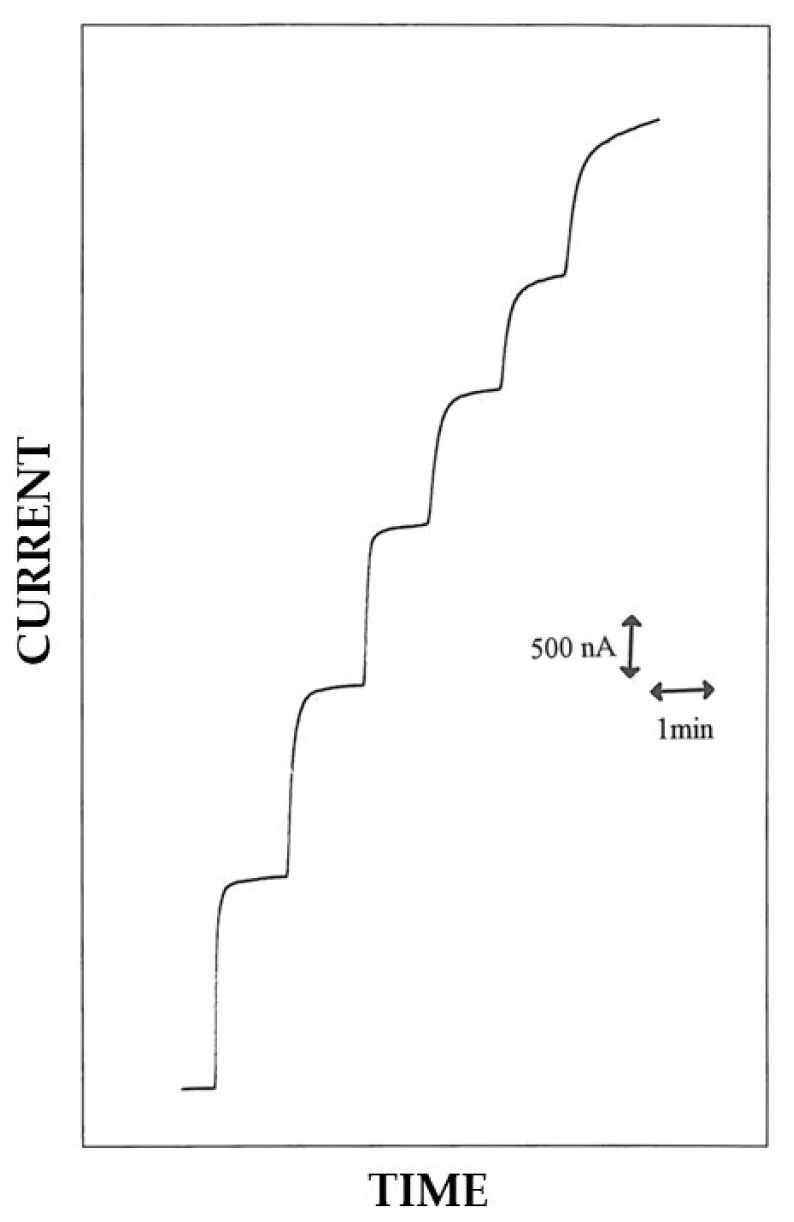
Typical current–time responses at a rotating disk Pt/GOD/P2NAP electrode for successive additions of glucose to an air-saturated phosphate buffer (pH 7, *I* 0.1 M). Glucose addition in the figure refers to total glucose concentrations of 1.25, 2.49, 3.72, 4.95, and 7.39 mM, and more. The disk rotation rate was 1000 rpm and the electrode diameter 2 mm; other conditions as described in the Materials and Methods section.

Accordingly, Figure 8 shows typical current–time responses for successive additions of glucose standard solutions on a Pt/GOD/P2NAP rotating disk electrode. Fast glucose responses were observed, with a response time *t*_0.95%_ less than 5 s in most cases. The relevant calibration curve, displaying the linear and saturated response at low and high glucose concentrations, respectively, as expected for enzyme catalysis, is shown in Figure S9 (upper plot). Also, for this last biosensor, significant deviations from the Michaelis–Menten model were observed as confirmed by the relevant Eadie–Hofstee graph (Figure S9, lower plot) which deviates from the expected linearity. Accordingly, diffusion of substrate through the enzymic layer became limiting, thus complicating the relevant kinetic behavior of biosensor. Such an interplay between enzyme and diffusional kinetics was confirmed also by a relevant hydrodynamic study reported in Figure S5. Even for Pt/GOD/P2NAP electrodes, the first portion of the curve showed an increase in the response with the rotation rate until reaching a steady state value at rotation rates higher than 1000 rpm, as expected for a membrane-modified electrode under diffusion control [28].

Table 1 reports the analytical performances of the present Pt/GOD/P2NAP electrode. Also in the present case, the sensitivity and maximal current were lower than the previously studied Pt/PoAP/GOD biosensor but almost similar to the Pt/GOD/PoAP case. Considering that all three cases shared, in the limit of the experimental error, the same enzyme membrane of comparable enzyme loading and thickness, this behavior confirmed that the exposure of the Pt/GOD electrode to the monomer bath for electrosynthesis of permselective layer may have been to some extent detrimental to GOD activity (*vide ante*). Lastly, the apparent Michaelis–Menten constant and the upper limit of the linear range were lower than the previous cases, supporting the diffusional constraints in its kinetic behavior. Also in the present case, hyperglycemic sample analysis would require sample dilution as well.

**Figure 9:**
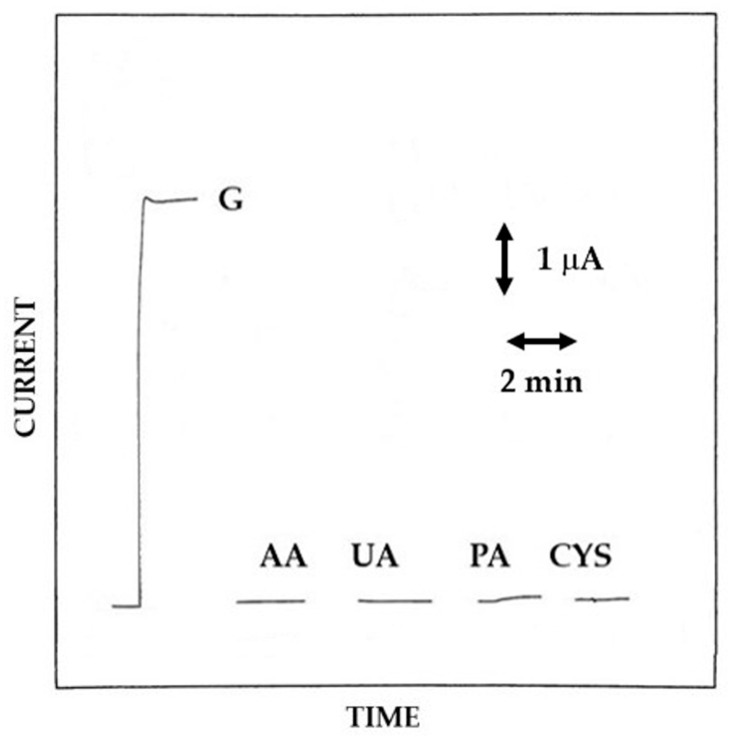
Typical current–time responses at a rotating disk Pt/GOD/P2NAP electrode for additions of glucose (G) 5 mM, ascorbic acid (AA) 0.1 mM, uric acid (UA) 0.5 mM, paracetamol (PA) 0.2 mM, and cysteine (CYS) 0.1 mM to an air-saturated phosphate buffer (pH 7, *I* 0.1 M). The disk rotation rate was 1000 rpm and the electrode diameter 2 mm; other conditions as described in the Materials and Methods section.

The anti-interference capability of the Pt/GOD/P2NAP biosensor is illustrated in Figure 9 which compares a typical glucose response at 5 mM level with those relevant to the already chosen interfering compounds (*vide ante*); Table 2 summarizes the relevant bias introduced in glucose measurement from these interfering species. As can be seen, all the interference responses were completely rejected and the relevant bias in glucose measurements were so low to be inestimable. These performances, quite promising for the use of Pt/GOD/P2NAP biosensors in real sample analysis, are in agreement with those already reported elsewhere [10,20], thus confirming the extreme rejection ability of electrosynthesized films from 2-naphtol. Notably, the observed features confirmed the usefulness of the approach described. Permselective film can be easily electrosynthesized even though an enzymic layer without losing their properties, at least for the P2NAP case.

Lastly, a preliminary storage stability study for the present Pt/GOD/P2NAP electrode was performed by monitoring the sensibility of these electrodes during their use and after storage in a buffer medium. After more than two weeks, the Pt/GOD/P2NAP biosensor dropped its sensitivity only 5% of its initial value, demonstrating a good storage stability. Further, the within-day (*n* = 3) and the between-days (*n* = 6) coefficient of variation for glucose response at 5 mM level were 4% and 15%, respectively.

## 4. Conclusions

The simple application of electrical potential to an electrode in an electrochemical cell mainly promotes two different phenomena which were altogether utilized in this study. The consequential establishing of an electrical field stimulates electrophoretic migration of charged particles which could encourage deposit formation onto the electrode surface. In the present case, this phenomena permitted co-crosslinking of glucose oxidase and bovine serum albumin (a classical immobilization procedure) exclusively onto the surface of the used transducer, without any use of external membranes incorporating enzymes and/or physical manipulations like casting [23]. The application of an electrical potential also changes the electron energy level of the electrode [30], i.e., the availability of electrons at the electrode interphase, promoting electron transfer with suitable species available in the electrochemical cell. This phenomenon permitted, in the present case, the in situ electrosynthesis and electrodeposition of a thin permselective membrane onto the electrode surface, increasing the selectivity of the transducer, thus permitting its used in real sample analysis. This study demonstrates that both these approaches can be used altogether as a new, original approach to produce amperometric enzyme electrodes.

Even if both approaches cannot be used simultaneously for the different potential stimuli they require, both can be used in sequential mode by producing at least glucose biosensors with good analytical performances and acceptable interferent rejection properties. Indeed, the preliminary electrophoretic protein deposition step permitting glucose oxidase immobilization followed by electrosynthesis of non-conducting, permselective film gave the best results in terms of enzyme deposit and permselectivity properties, particularly for the case of poly-2-naphthol films, thus permitting the fabrication of a fast-response glucose sensor with good sensitivity and unbeatable interferent rejection, surely promising for real sample analysis applications. Please note that the use and the proper coupling of these two electrochemical procedures were never explored and reported before, so this study would represent the first report regarding this all-electrochemically assisted procedure for biosensor production.

Nowadays, biosensor fabrication relies on various techniques to produce sensors with high sensitivity, selectivity, and rapid response times. These methods, involving electrode fabrication, bio-recognition element immobilization, and transducer integration, embrace microfabrication, screen, and inkjet (bio)printing technologies and nanomaterial integration [31]. We feel that the present all-electrochemically approach, like the classical electroplating procedures, permits an accurate bi-dimensional control of enzyme immobilization and permselective film formation onto the electrodic sensor, an easy preparation of biosensor, and the possibility of miniaturization of the biosensing devices, and can surely play a role in this context as well.

## Figures and Tables

**Table 1:** Analytical performances of biosensors ^1^.

Biosensor	Sensitivity (µA/mM mm^2^)	*I*_max_ ^2^ (µA/mm^2^)	LD/LQ ^3^ (µM)	Linear Range (mM)	*K*′_m_ ^4^ (mM)
Pt/PoAP/GOD	0.60	10.8	55/180	0.1–8	13.4
Pt/GOD/PoAP	0.37	4.29	96/312	0.2–4	6.33
Pt/GOD/P2NAP	0.33	3.31	48/156	0.1–3	4.45

^1^ Supporting electrolyte: air-saturated phosphate buffer (pH 7, *I* 0.1 M); disk rotation rate 1000 rpm, electrode diameter 2 mm; other conditions as described in the Materials and Methods section. ^2^ *I*_max_: maximal current density. ^3^ LD: limit of detection; LQ: limit of quantification. ^4^ *K*′_m_: apparent Michaelis–Menten constant.

**Table 2:** Bias in 5 mM glucose measurements from typical interfering compounds ^1^.

Biosensor	Bias (%) ^1^			
	AA ^2^ (0.1 mM)	UA ^2^ (0.5 mM)	PA ^2^ (0.2 mM)	CYS ^2^ (0.1 mM)
Pt/PoAP/GOD	3.85	16.9	5.38	3.46
Pt/GOD/PoAP	1.42	2.48	10.3	3.55
Pt/GOD/P2NAP	NV ^3^	NV ^3^	NV ^3^	NV ^3^

^1^ The bias percentage was calculated by multiplying by 100, the ratio of interference response (at the selected concentration) to glucose response (valuated at a 5 mM concentration). ^2^ Legend of acronyms: AA ascorbic acid, UA uric acid, PA paracetamol, and CYS cysteine; the bias for each compound was evaluated at the shown concentration level. ^3^ NV: not valuable since the interference response was almost zero. Supporting electrolyte: air-saturated phosphate buffer (pH 7, *I* 0.1 M); disk rotation rate 1000 rpm, electrode diameter 2 mm; other conditions as described in the Materials and Methods section.

## Data Availability

The original contributions presented in this study are included in the article. Further inquiries can be directed to the corresponding author.

## Supplementary Materials

The following supporting information can be downloaded at: https://www.mdpi.com/article/10.3390/bios15080470/s1. Scheme S1. Schematic representation of a biosensor. From left to right: the sample containing the target analyte (in the middle), the biosensor consisting of a biocomponent (displayed in white) properly associated with the transducer (displayed in grey), and the relevant generated signal (arrow). Scheme S2. Simplified representation of the analyte detection approach in enzyme amperometric biosensors and their detection weaknesses in real sample analysis. The target analyte, i.e., the enzyme substrate in its reduced form S_RED_, reacts with the enzyme (ENZ, an oxidoreductase) producing dihydrogen peroxide which is promptly oxidized at the electrode, generating a current proportional to the substrate concentration. The presence in the sample of interfering electroactive compounds (here *I*) and surface-active substances (here SAS) like high molecular weight proteins could bias and eventually totally hamper the dihydrogen peroxide amperometric detection, respectively. Scheme S3. The approach in biosensor fabrication for conventional enzyme amperometric sensors. The top membrane (e.g., polyurethane) prevents high molecular weight, surface active substance (SAS) (e.g., albumin) from reaching and fouling the electrode surface while assuring diffusion of target analyte, i.e., the enzyme substrate (S), and cofactor, i.e., 0_2_. The middle membrane is required for enzyme immobilization and consists of various materials depending on the immobilization procedure. The bottom membrane (e.g., cellulose acetate) onto the top of electrode surface prevents undesired sensing of endogenous interfering electroactive compounds (I) and hence bias or other undesired effects in analyte detection. Scheme S4. Schematic representation of a biosensor based on enzyme entrapment into electrosynthesized polymers. During the in situ electrosynthesis of the polymers onto the electrode surface, the enzyme molecules (in yellow) remain entrapped and hence immobilized onto the electrochemical transducer. In the case of permselective, non-conducting polymers, the entrapping polymeric film also performs as an antifouling and permselective membrane, reducing the undesired effects due to high molecular weight, surface active substance (SAS), and endogenous interfering electroactive compounds (I). Scheme S5. The “*hybrid*” approach for biosensor fabrication, here drafted as an enzyme membrane (produced by the co-crosslinking of the enzyme molecules (ENZ) with an inert protein such as bovine serum albumin (BSA) through a crosslinker like glutaraldehyde (GLU)) onto the top of an electrosynthesized polymer-modified electrode. Scheme S6. Schematic representation of the current pulse sequence applied at the deposition electrode in galvanodynamic experiments. *I* represents the maximal current value of the pulse, *t*_1_ the pulse inactive, and *t*_2_ the pulse active time, i.e., the pulse width of the waveform. Figure S1. Typical cyclic voltammograms of potassium ferricyanide 5 mM (a) and hexaammineruthenium (III) chloride 3.2 mM (b) in phosphate buffer (pH 7, *I* 0.1 M) on bare Pt electrodes (continuous lines) and Pt-modified electrodes with the enzyme layer (dotted lines). The scan rate was 50 mV/s and the electrode diameter 3 mm; other conditions as described in the Materials and Methods section. Figure S2. Typical cyclic voltammograms of potassium ferricyanide 5 mM in phosphate buffer (pH 7, *I* 0.1 M) on bare Pt electrode (a), Pt-modified electrodes with poly-2-naphtol (P2NAP) (b) and poly-o-aminophenol (PoAP) (c). The scan rate was 50 mV/s and the electrode diameter 3 mm; other conditions as described in the Materials and Methods section. Figure S3. Typical cyclic voltammograms of a bare (continuous line) and poly-o-aminophenol (PoAP)-modified Pt electrode (dotted line) in phosphate buffer (pH 7, *I* 0.1 M). The scan rate was 50 mV/s and the electrode diameter 3 mm; other conditions as described in the Materials and Methods section. Figure S4. Calibration curve (upper plot) and Eadie–Hofstee plot of calibration curve data (lower plot) for a typical rotating disk Pt/PoAP/GOD electrode. Continuous line in the upper plot refers to Michaelis–Menten fitting of data. All experimental conditions were those described in Figure 4. Figure S5. Normalized steady-state current responses of different biosensors (see legend) at several rotation rates due to the addition of glucose standard solution 1.5 mM to an air-saturated phosphate buffer (pH 7, *I* 0.1 M). Electrode diameter 2 mm; other conditions as described in the Materials and Methods section. Figure S6. Normalized sensitivity to glucose for a rotating disk Pt/PoAP/GOD electrode stored in a phosphate buffer (pH 7, *I* 0.1 M) at 4 °C in the dark when not in use. Electrode diameter 2 mm; other conditions as described in the Materials and Methods section. Figure S7. Calibration curve (upper plot) and Eadie–Hofstee plot of calibration curve data (lower plot) for a typical rotating disk Pt/GOD/PoAP electrode. Continuous line in the upper plot refers to Michaelis–Menten fitting of data. All experimental conditions were those described in Figure 6. Figure S8. Normalized sensitivity to glucose for a rotating disk Pt/GOD/PoAP electrode stored in a phosphate buffer (pH 7, *I* 0.1 M) at 4 °C in the dark when not in use. Electrode diameter 2 mm; other conditions as described in the Materials and Methods section. Figure S9. Calibration curve (upper plot) and Eadie–Hofstee plot of calibration curve data (lower plot) for a typical rotating disk Pt/GOD/P2NAP electrode. The continuous line in upper plot refers to Michaelis–Menten fitting of data. All experimental conditions were those described in Figure 8.

## References

[1] Owen V.M., Turner A.P.F. (1987). Biosensors: A revolution in clinical analysis?. Endeavour.

[2] Turner A.P.F. (1989). Current trends in biosensor research and development. Sens. Actuators.

[3] Clark L.C., Lyons C. (1962). ELECTRODE SYSTEMS FOR CONTINUOUS MONITORING IN CARDIOVASCULAR SURGERY. Ann. N. Y. Acad. Sci..

[4] Bollella P. (2022). Enzyme-based amperometric biosensors: 60 years later … Quo Vadis?. Anal. Chim. Acta.

[5] Kalita N., Gogoi S., Minteer S.D., Goswami P. (2023). Advances in Bioelectrode Design for Developing Electrochemical Biosensors. ACS Meas. Sci. Au.

[6] Cass A.E.G. (1990). Biosensors. A Practical Approach.

[7] Bartlett P.N., Cooper J.M. (1993). A review of the immobilization of enzymes in electropolymerized films. J. Electroanal. Chem..

[8] Malitesta C., Palmisano F., Torsi L., Zambonin P.G. (1990). Glucose fast-response amperometric sensor based on glucose oxidase immobilized in an electropolymerized poly(o-phenylenediamine) film. Anal. Chem..

[9] Bartlett P.N., Tebbutt P., Tyrrell C.H. (1992). Electrochemical immobilization of enzymes. 3. Immobilization of glucose oxidase in thin films of electrochemically polymerized phenols. Anal. Chem..

[10] Ciriello R., Cataldi T.R.I., Centonze D., Guerrieri A. (2000). Permselective Behavior of an Electrosynthesized, Nonconducting Thin Film of Poly(2-naphthol) and Its Application to Enzyme Immobilization. Electroanalysis.

[11] Guerrieri A., Ciriello R., Centonze D. (2009). Permselective and enzyme-entrapping behaviours of an electropolymerized, non-conducting, poly(o-aminophenol) thin film-modified electrode: A critical study. Biosens. Bioelectron..

[12] Palmisano F., Centonze D., Guerrieri A., Zambonin P.G. (1993). An interference-free biosensor based on glucose oxidase electrochemically immobilized in a non-conducting poly(pyrrole) film for continuous subcutaneous monitoring of glucose through microdialysis sampling. Biosens. Bioelectron..

[13] Pantano P., Kuhr W.G. (1995). Enzyme-modified microelectrodes for in vivo neurochemical measurements. Electroanalysis.

[14] Bartlett P.N., Whitaker R.G. (1987). Electrochemical immobilisation of enzymes: Part II. Glucose oxidase immobilised in poly-N-methylpyrrole. J. Electroanal. Chem. Interfacial Electrochem..

[15] De Benedetto G.E., Malitesta C., Zambonin C.G. (1994). Electroanalytical/X-ray photoelectron spectroscopy investigation on glucose oxidase adsorbed on platinum. J. Chem. Soc. Faraday Trans..

[16] Sasso S.V., Pierce R.J., Walla R., Yacynych A.M. (1990). Electropolymerized 1, 2-Diaminobenzene as a Means To Prevent Interferences and Fouling and To Stabilize Immobilized Enzyme in Electrochemical Biosensors. Anal. Chem..

[17] Geise R.J., Adams J.M., Barone N.J., Yacynych A.M. (1991). Electropolymerized films to prevent interferences and electrode fouling in biosensors. Biosens. Bioelectron..

[18] Kennedy J.F., White C.A., Wiseman A. (1985). Principles of immobilization of enzymes. Handbook of Enzyme Biotechnology.

[19] Guerrieri A., De Benedetto G.E., Palmisano F., Zambonin P.G. (1998). Electrosynthesized non-conducting polymers as permselective membranes in amperometric enzyme electrodes: A glucose biosensor based on a co-crosslinked glucose oxidase/overoxidized polypyrrole bilayer. Biosens. Bioelectron..

[20] Guerrieri A., Lattanzio V., Palmisano F., Zambonin P.G. (2006). Electrosynthesized poly(pyrrole)/poly(2-naphthol) bilayer membrane as an effective anti-interference layer for simultaneous determination of acethylcholine and choline by a dual electrode amperometric biosensor. Biosens. Bioelectron..

[21] Guerrieri A., Ciriello R., Cataldi T.R.I. (2013). A novel amperometric biosensor based on a co-crosslinked l-lysine-α-oxidase/overoxidized polypyrrole bilayer for the highly selective determination of l-lysine. Anal. Chim. Acta.

[22] Ciriello R., Guerrieri A. (2021). A Crosstalk- and Interferent-Free Dual Electrode Amperometric Biosensor for the Simultaneous Determination of Choline and Phosphocholine. Sensors.

[23] Guerrieri A., Ciriello R., Acquavia M.A., Bianco G., Di Capua A. (2023). Electrophoretic Protein Deposition as a Tool for In Situ Co-Crosslinking Enzyme Immobilization: An Electrochemical/Quartz Crystal Microbalance Study. Appl. Sci..

[24] Lacefield W.R. (1998). Current Status of Ceramic Coatings for Dental Implants. Implant Dent..

[25] Boccaccini A.R., Keim S., Ma R., Li Y., Zhitomirsky I. (2010). Electrophoretic deposition of biomaterials. J. R. Soc. Interface.

[26] Yang Q., Atanasov P., Wilkins E. (1998). Development of needle-type glucose sensor with high selectivity. Sensors Actuators B Chem..

[27] Zaborsky O. (1973). Immobilized Enzymes.

[28] Gough D.A., Leypoldt J.K. (1979). Membrane-covered, rotated disk electrode. Anal. Chem..

[29] Levich V.G. (1962). Physicochemical Hydrodynamics.

[30] Allen J., Bard L.R.F. (2000). Electrochemical Methods: Fundamentals and Applications.

[31] Sadana A., Sadana N., Sadana A., Sadana N. (2011). Fabrication of Biosensors. Handbook of Biosensors and Biosensor Kinetics.

