# Supplementary material for: A New Approach for Interferent-Free Amperometric Biosensor Production Based on All-Electrochemically Assisted Procedures
Source: Biosensors (Basel). 2025 Jul 22;15(8):470. doi: 10.3390/bios15080470 (PMC12384068; doi:10.3390/bios15080470)
Supplement: Supplementary file 1 [file biosensors-15-00470-s001.zip › biosensors-3737597-supplementary.pdf]

biosensors

Supplementary Materials

A New Approach for Interferent-Free Amperometric Biosensor Production Based on All-Electrochemically Assisted Procedures

Rosanna Ciriello, Maria Assunta Acquavia, Giuliana Bianco, Angela Di Capua and Antonio Guerrieri*

Dipartimento di Scienze di Base e Applicate, Università degli Studi della Basilicata, Via dell'Ateneo Lucano 10, 85100 Potenza, Italy
* Correspondence: antonio.guerrieri@unibas.it

Citation: To be added by editorial staff during production.

Received: date
Revised: date
Accepted: date
Published: 22 July 2025

Copyright: © 2025 by the authors. Licensee MDPI, Basel, Switzerland. This article is an open access article distributed under the terms and conditions of the Creative Commons Attribution (CC BY) license (https://creativecommons.org/licenses/by/4.0/).

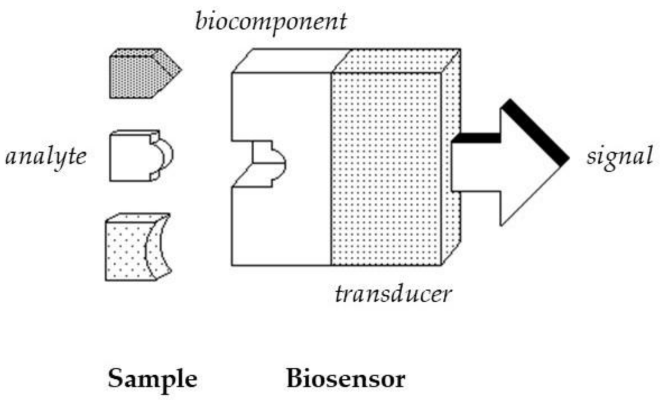

Scheme S1. Schematic representation of a biosensor. From left to right: the sample containing the target analyte (in the middle), the biosensor consisting of a biocomponent (displayed in white) properly associated with the transducer (displayed in grey) and the relevant generated signal (arrow).

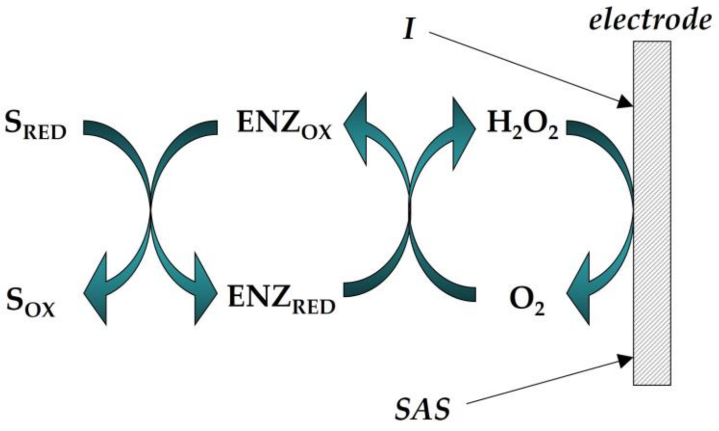

Scheme S2. Simplified representation of the analyte detection approach in enzyme amperometric biosensors and their detection weaknesses in real sample analysis. The target analyte, *i.e.* the enzyme substrate in its reduced form S_{RED}, reacts with the enzyme (ENZ, here an oxidoreductase) producing dihydrogen peroxide which is promptly oxidized at the electrode, generating a current proportional to the substrate concentration. The presence in the sample of interfering electroactive compounds (here *I*) and surface-active substances (here **SAS**) like high molecular weight proteins could bias and eventually totally hamper the dihydrogen peroxide amperometric detection, respectively.

 https://doi.org/10.3390/bios15080470

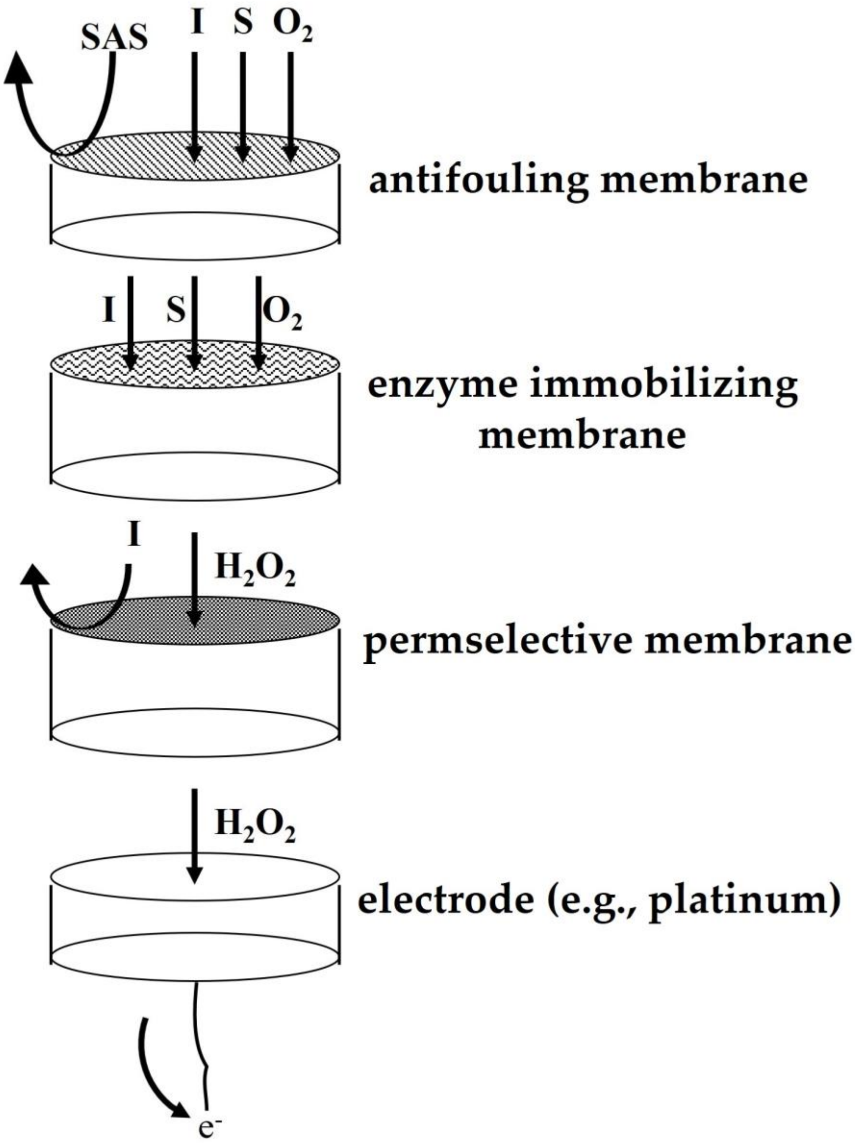

Scheme S3. The approach in biosensor fabrication for conventional enzyme amperometric sensors. The top membrane (*e.g.* polyurethane) prevents high molecular weight, surface active substance (SAS) (*e.g.* albumin) from reaching and fouling the electrode surface while assuring diffusion of target analyte, *i.e.* the enzyme substrate (S), and cofactor, *i.e.* 0_2. The middle membrane is required for enzyme immobilization and consists of various materials depending on the immobilization procedure. The bottom membrane (*e.g.* cellulose acetate) onto the top of electrode surface prevents undesired sensing of endogenous interfering electroactive compounds (I) and hence bias or other undesired effects in analyte detection.

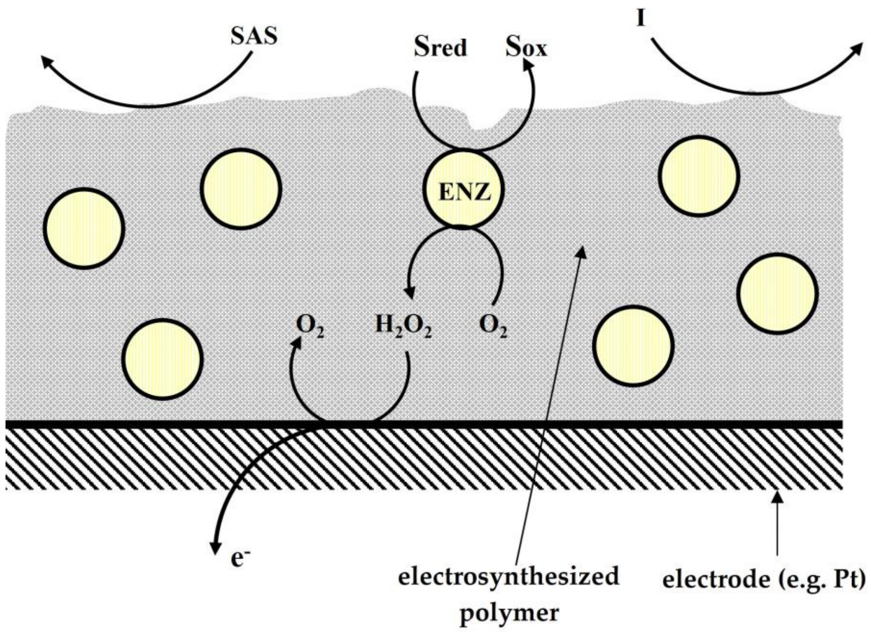

Scheme S4. Schematic representation of a biosensor based on enzyme entrapment into electrosynthesized polymers. During the in-situ electrosynthesis of the polymers onto the electrode surface, the enzyme molecules (in yellow) remain entrapped and hence immobilized onto the electrochemical transducer. In the case of permselective, non-conducting polymers, the entrapping polymeric film also performs as an antifouling and permselective membrane reducing the undesired effects due to high molecular weight, surface active substance (SAS) and endogenous interfering electroactive compounds (I).

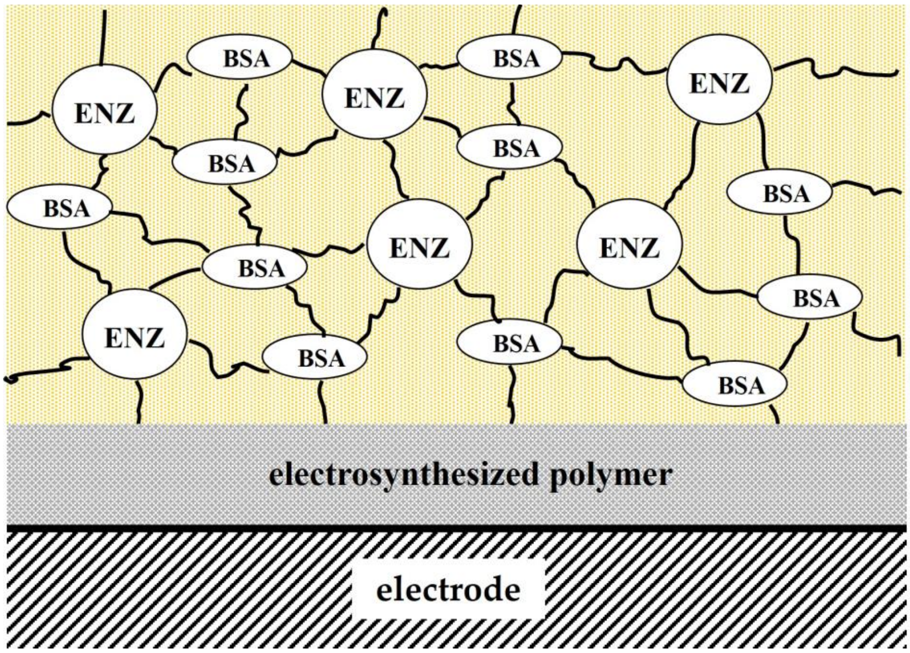

Scheme S5. The "*hybrid*" approach for biosensor fabrication, here drafted as an enzyme membrane (produced by the co-crosslinking of the enzyme molecules (ENZ) with an inert protein such as bovine serum albumin (BSA) through a crosslinker like glutaraldehyde (GLU)) onto the top of an electrosynthesized polymer modified electrode.

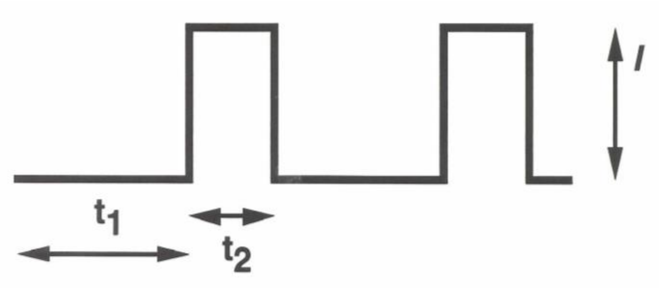

Scheme S6. Schematic representation of the current pulse sequence applied at the deposition electrode in galvanodynamic experiments. *I* represent the maximal current value of the pulse, t_1 the pulse inactive and t_2 the pulse active time, *i.e.*, the pulse width of the waveform.

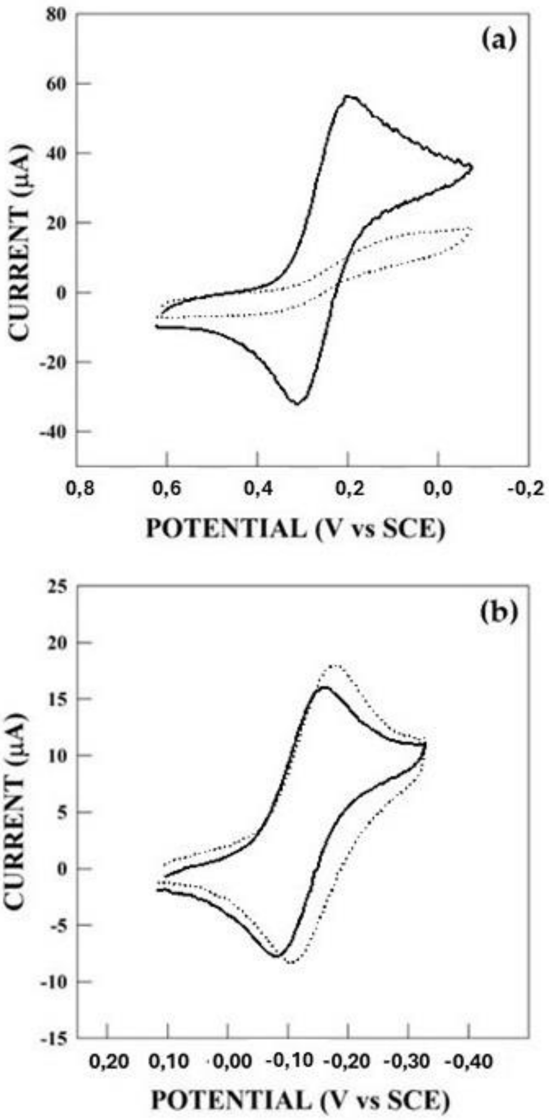

Figure S1. Typical cyclic voltammograms of potassium ferricyanide 5 mM (a) and hexaammineruthenium (III) chloride 3.2 mM (b) in phosphate buffer (pH 7, *I* 0.1 M) on bare Pt electrodes (continuous lines) and Pt modified electrodes with the enzyme layer (dotted lines). Scan rate was 50 mV/s and electrode diameter 3 mm; other conditions as described in the Materials and Methods section.

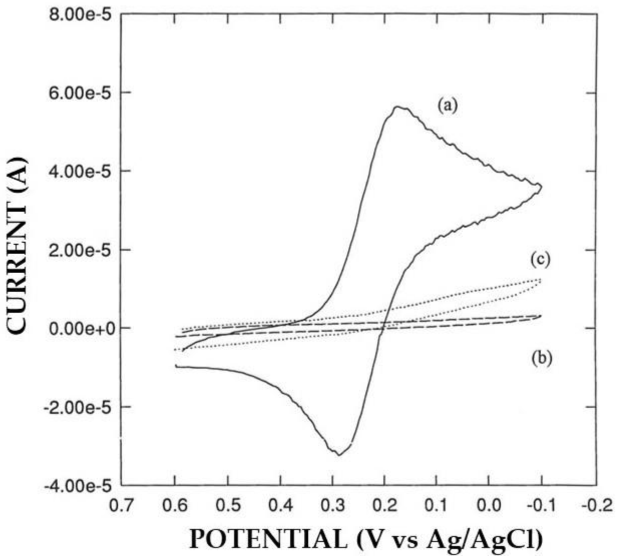

Figure S2. Typical cyclic voltammograms of potassium ferricyanide 5 mM in phosphate buffer (pH 7, *I* 0.1 M) on bare Pt electrode (a), Pt modified electrodes with poly-2-naphtol (P2NAP) (b) and poly-o-aminophenol (PoAP) (c). Scan rate was 50 mV/s and electrode diameter 3 mm; other conditions as described in the Materials and Methods section.

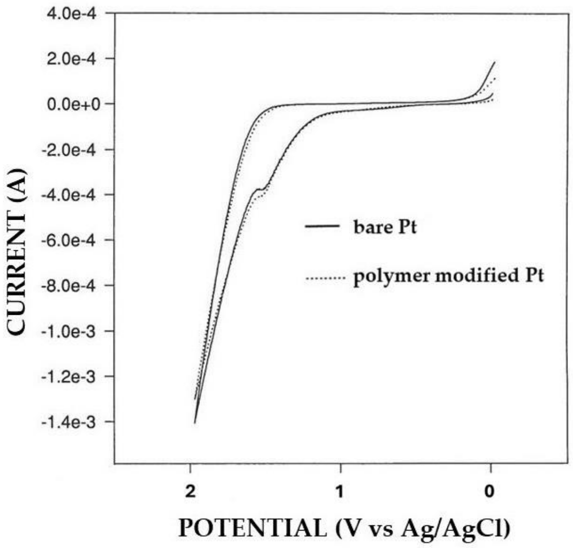

Figure S3. Typical cyclic voltammograms of a bare (continuous line) and poly-o-aminophenol (PoAP) modified Pt electrode (dotted line) in phosphate buffer (pH 7, *I* 0.1 M). Scan rate was 50 mV/s and electrode diameter 3 mm; other conditions as described in the Materials and Methods section.

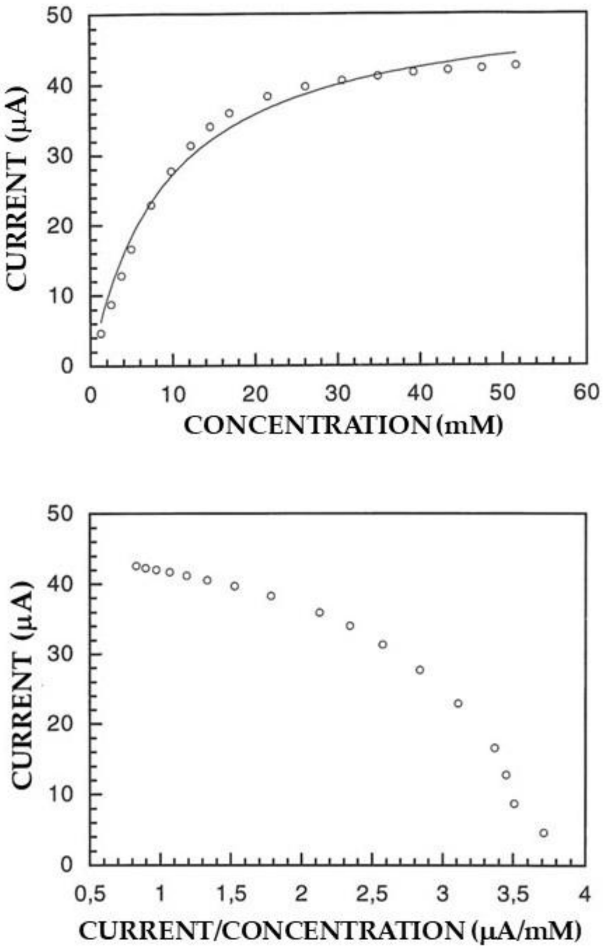

Figure S4. Calibration curve (upper plot) and Eadie-Hofstee plot of calibration curve data (lower plot) for a typical rotating disk Pt/PoAP/GOD electrode. Continuous line in upper plot refers to Michaelis-Menten fitting of data. All experimental conditions were those described in Figure 4.

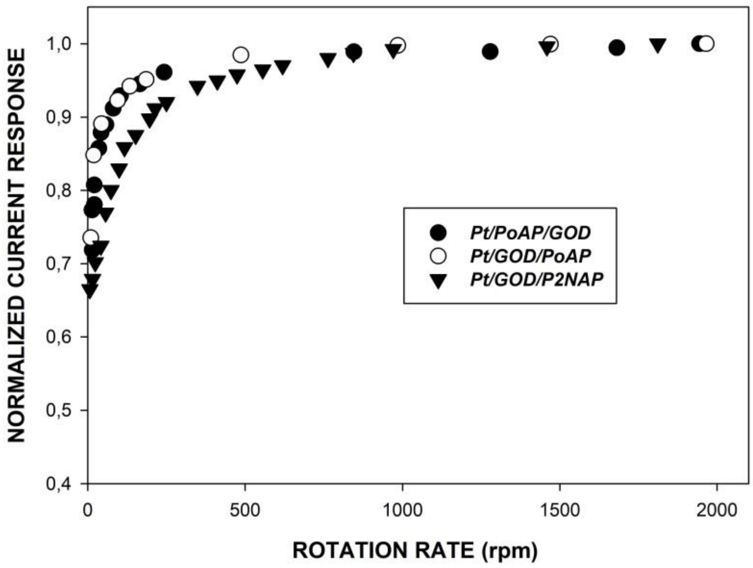

Figure S5. Normalized steady-state current responses of different biosensors (see legend) at several rotation rates due to the addition of glucose standard solution 1.5 mM to an air saturated phosphate buffer (pH 7, *I* 0.1 M). Electrode diameter 2 mm; other conditions as described in the Materials and Methods section.

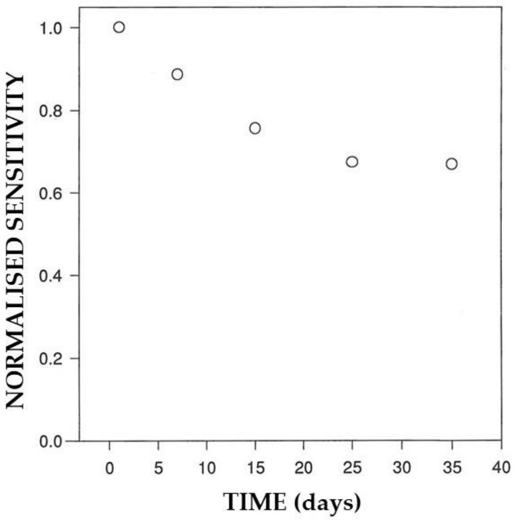

Figure S6. Normalised sensitivity to glucose for a rotating disk Pt/PoAP/GOD electrode stored in a phosphate buffer (pH 7, *I* 0.1 M) at 4° C in the dark when not in use. Electrode diameter 2 mm; other conditions as described in the Materials and Methods section.

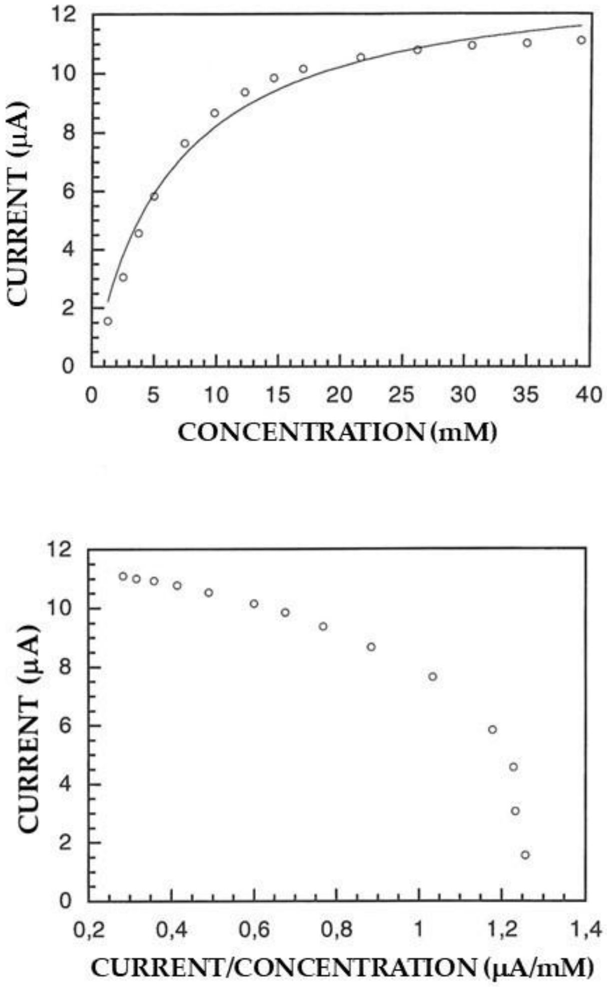

Figure S7. Calibration curve (upper plot) and Eadie-Hofstee plot of calibration curve data (lower plot) for a typical rotating disk Pt/GOD/PoAP electrode. Continuous line in upper plot refers to Michaelis-Menten fitting of data. All experimental conditions were those described in Figure 6.

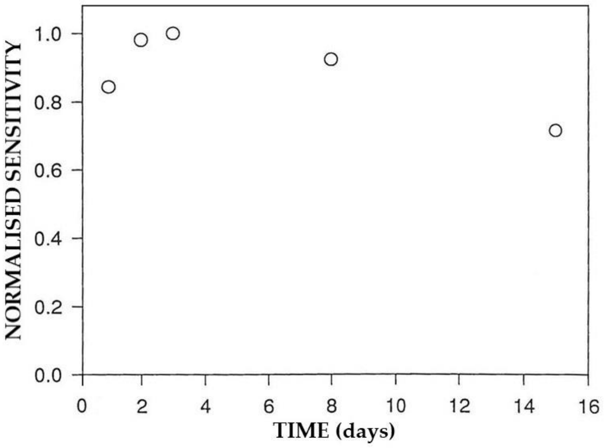

Figure S8. Normalised sensitivity to glucose for a rotating disk Pt/GOD/PoAP electrode stored in a phosphate buffer (pH 7, *I* 0.1 M) at 4° C in the dark when not in use. Electrode diameter 2 mm; other conditions as described in the Materials and Methods section.

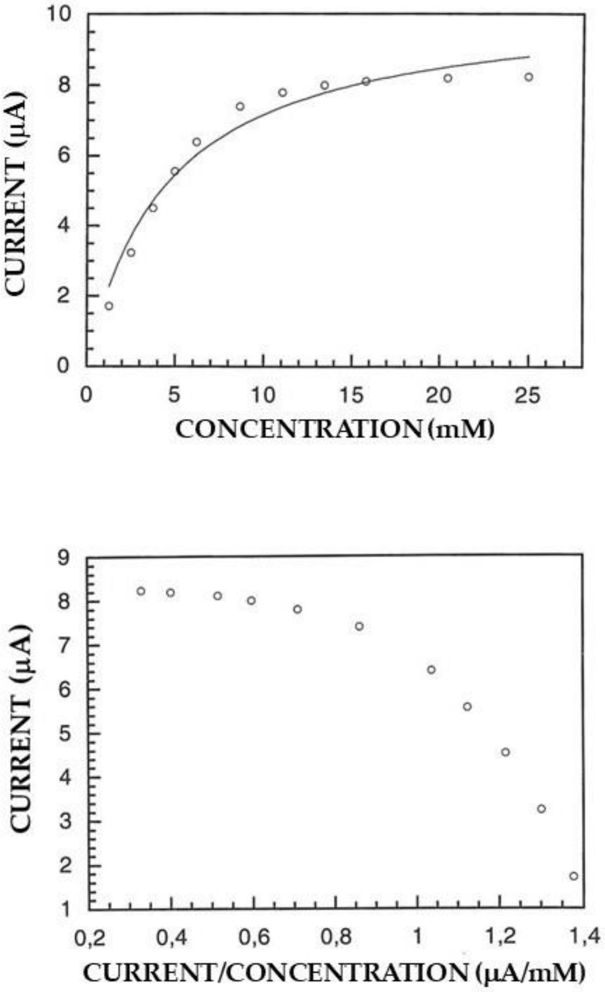

Figure S9. Calibration curve (upper plot) and Eadie-Hofstee plot of calibration curve data (lower plot) for a typical rotating disk Pt/GOD/P2NAP electrode. Continuous line in upper plot refers to Michaelis-Menten fitting of data. All experimental conditions were those described in Figure 8.
